# Ovarian mature cystic teratoma with fistula formation into the rectum: a case report

**DOI:** 10.1186/s40064-016-3426-4

**Published:** 2016-10-03

**Authors:** Yuichiro Kizaki, Tomonori Nagai, Ken Ohara, Yosuke Gomi, Taichi Akahori, Yoshihisa Ono, Shigetaka Matsunaga, Yasushi Takai, Masahiro Saito, Kazunori Baba, Hiroyuki Seki

**Affiliations:** Department of Obstetrics and Gynecology, Saitama Medical Center, Saitama Medical University, 1981 Kamoda, Kawagoe-shi, Saitama Japan

**Keywords:** Diarrhea, Fistula, Inflammation, Ovarian neoplasms, Teratoma

## Abstract

**Background:**

While ovarian mature cystic teratomas are benign ovarian germ-cell tumors and the most common type of all ovarian tumors, the formation of fistulas into surrounding organs such as the bladder and the intestinal tract is extremely rare. This report documents a case of ovarian mature cystic teratoma with a rectal fistula, thought to be caused by local inflammation.

**Case description:**

A pelvic mass was diagnosed as an ovarian mature cystic teratoma of approximately 10 cm in diameter on transvaginal ultrasound and magnetic resonance examinations. Endoscopic examination of the lower gastrointestinal tract to investigate diarrhea revealed an ulcerative lesion with hair in the rectal wall adjacent to the ovarian cyst, and formation of a fistula from the ovarian teratoma into the rectum was suspected. Laparotomy revealed extensive inflammatory adhesions between a left ovarian tumor and the rectum. Left salpingo-oophorectomy and upper anterior resection of the rectum were performed. The final pathological diagnosis was ovarian mature cystic teratoma with no malignant findings, together with severe rectal inflammation and fistula formation with no structural disorders such as diverticulitis of the colon or malignant signs.

**Discussion:**

The formation of fistulas and invasion into the neighboring organs are extremely rare complications for ovarian mature cystic teratomas. The invasion of malignant cells into neighboring organs due to malignant transformation of the tumor is reported as the cause of fistula formation into the neighboring organs. A review of 17 cases including the present case revealed that fistula formation due to malignant transformation comprised only 4 cases (23.5 %), with inflammation as the actual cause in the majority of cases (13 cases, 76.5 %).

**Conclusion:**

Although malignancy is the first consideration when fistula formation is observed between ovarian tumors and surrounding organs, in mature cystic teratoma, local inflammation is more likely than malignant transformation.

## Background

Ovarian mature cystic teratomas are benign ovarian germ-cell tumors and the most common type of all ovarian tumors, comprising 10–25 % of cases (Tandon et al. 2010). The most common known complication is torsion of the tumor, reportedly observed in approximately 16 % of cases (Tandon et al. 2010; Park et al. 2008). Meanwhile, formation of fistulas into neighboring organs is an extremely rare complication, occurring in less than 1 % of cases (Tandon et al. 2010; von-Walter 2012) and seldom reported. In the present case, endoscopy of the lower gastrointestinal tract performed to investigate refractory diarrhea resulted in the incidental discovery of a rectal fistula originating from an ovarian mature cystic teratoma.

## Case description

**Fig. 1:**
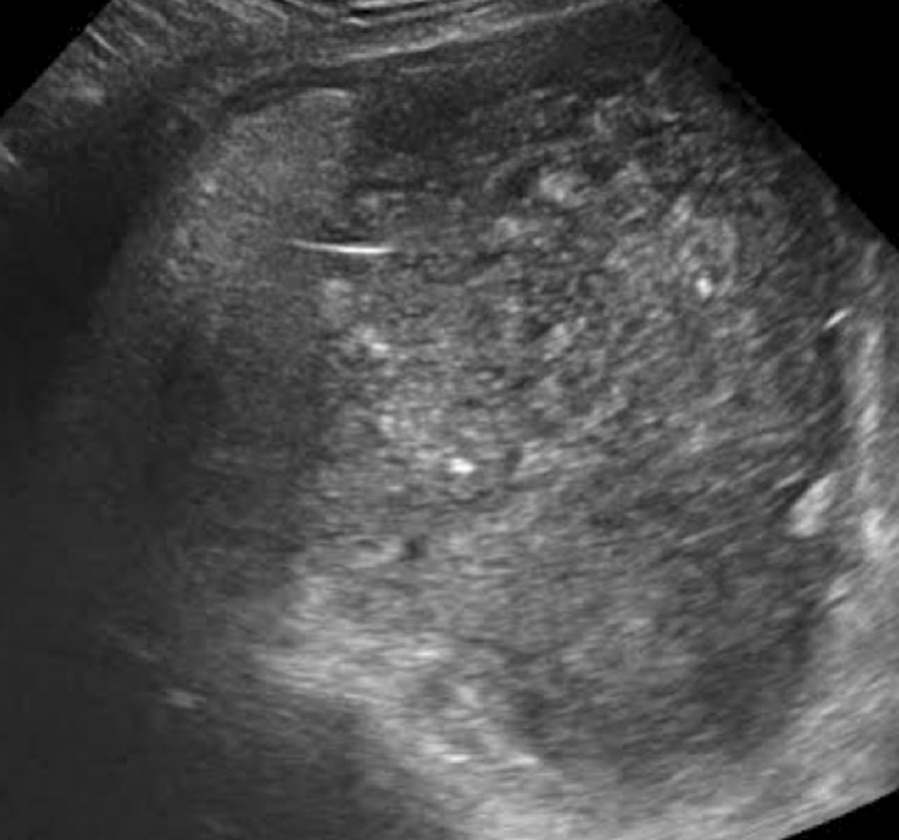
Transvaginal ultrasound image. A pelvic mass is seen of approximately 11 cm in diameter and is suspected to contain hair because of the presence of hyperechoic lines

The patient in this case was a 43-year-old woman. She had no significant past medical history and no familial history such as cancer and inflammatory bowel disease. She was examined by her local physician because of flu-like symptoms, fever, and diarrhea. Abdominal ultrasound examination incidentally revealed a pelvic mass, and she was referred to our institution. Transvaginal ultrasound examination revealed an 11 cm pelvic mass (Fig. 1). Inside the mass, suspected hair was observed in the form of hyperechoic lines, leading to suspicion of an ovarian mature cystic teratoma. Blood and biochemical tests did not reveal blood-count or other abnormalities. Serum levels of tumor markers including carcinoembryonic antigen (CEA; 1.0 ng/mL), carbohydrate antigen 125 (CA125; 59 U/mL), and carbohydrate antigen 19–9 (CA19–9; 9 U/mL) were within normal range, but that for squamous cell carcinoma antigen (SCC; 2.0 ng/mL) was elevated. Abdominal contrast-enhanced computed tomography (CT) revealed an 11 cm cystic tumor in the pelvis, containing lipids and a fat–fluid level. No distinct abnormalities were observed in other organs.

**Fig. 2:**
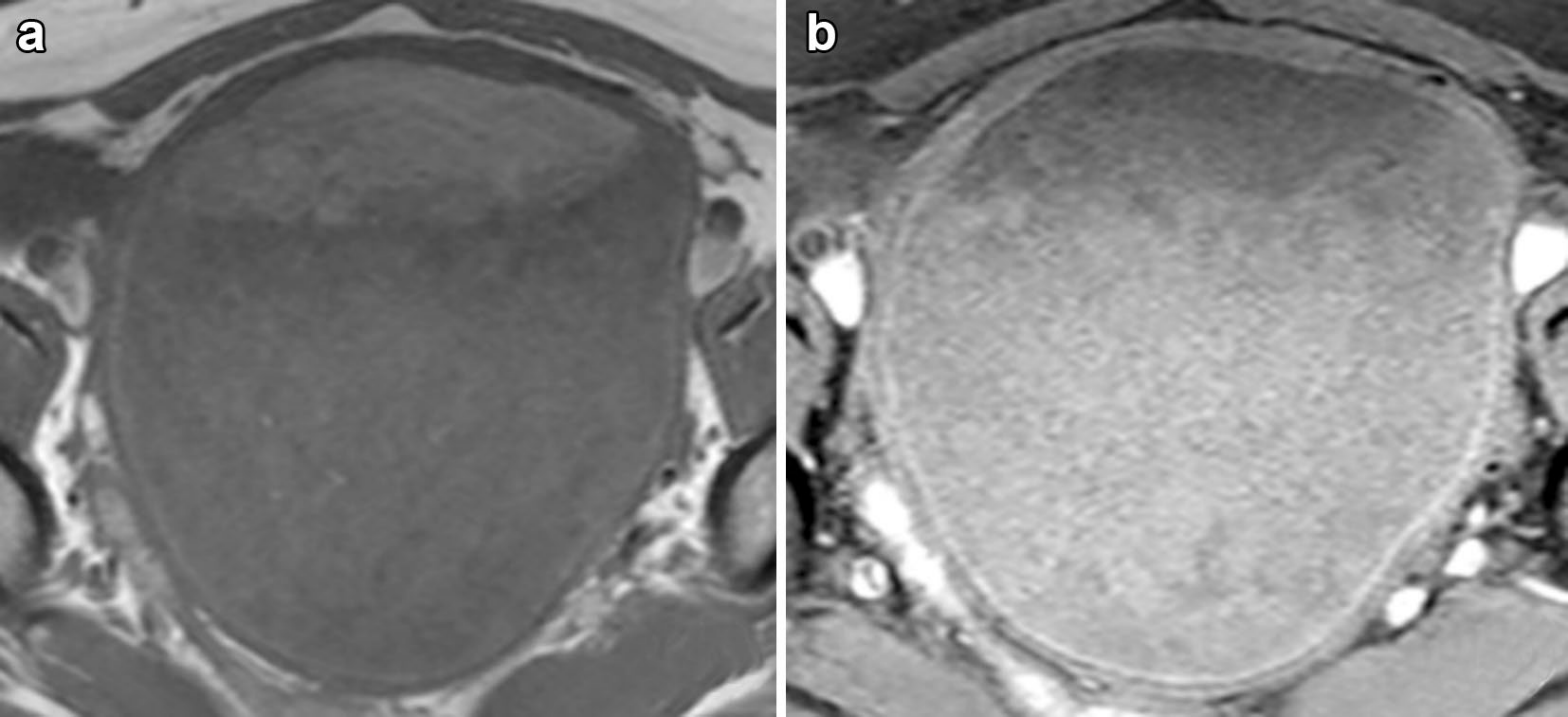
Magnetic resonance images. **a** A T1-weighted image shows a cystic tumor containing a fat–fluid level. **b** A fat-suppression image reveals fat suppression on the abdominal side of the niveau

The fat–fluid level inside the tumor was also observed on pelvic contrast-enhanced magnetic resonance imaging (MRI), with high signal intensity on the abdominal side in both T1- and T2-weighted images and low signal intensity on fat-suppression images, suggesting ovarian mature cystic teratoma (Fig. 2a, b). No solid areas with contrast enhancement to suggest malignancy were observed inside the tumor. From the imaging findings, an ovarian mature cystic teratoma was suspected and the decision was made to perform laparoscopic surgery.

**Fig. 3:**
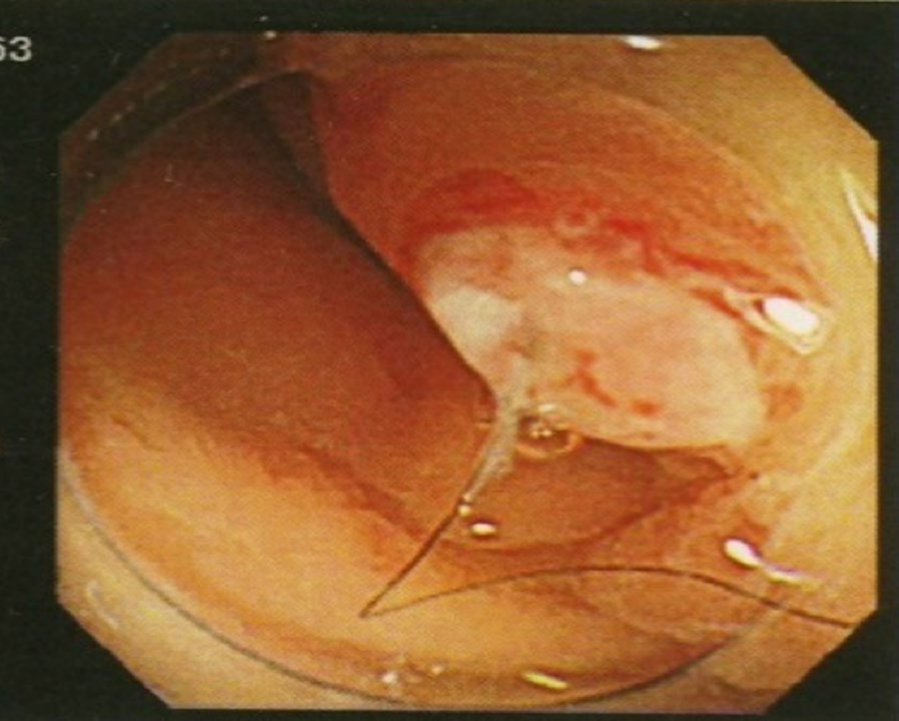
Lower gastrointestinal tract endoscopy image. Hair and a submucosal tumor-like protrusion with redness at its apex are seen

The patient also consulted the local medical gastroenterology clinic because of ongoing symptoms of diarrhea ranging in color from white to bright green. Endoscopic examination of the lower gastrointestinal tract revealed hair and a submucosal tumor-like protrusion with redness at its apex, situated approximately 12 cm adoral to the anal orifice (Fig. 3). No signs of diverticulitis were observed in the surrounding area. As this lesion site was adjacent to the ovarian tumor, and hair was present at the lesion site, formation of a fistula from the ovarian mature cystic teratoma into the rectum was suspected. As rectal invasion by a benign mature cystic teratoma is very unlikely, the possibility of malignant transformation of the mature cystic teratoma was considered, and the decision was made to perform salpingo-oophorectomy of the diseased side via laparotomy rather than laparoscopic surgery.

**Fig. 4:**
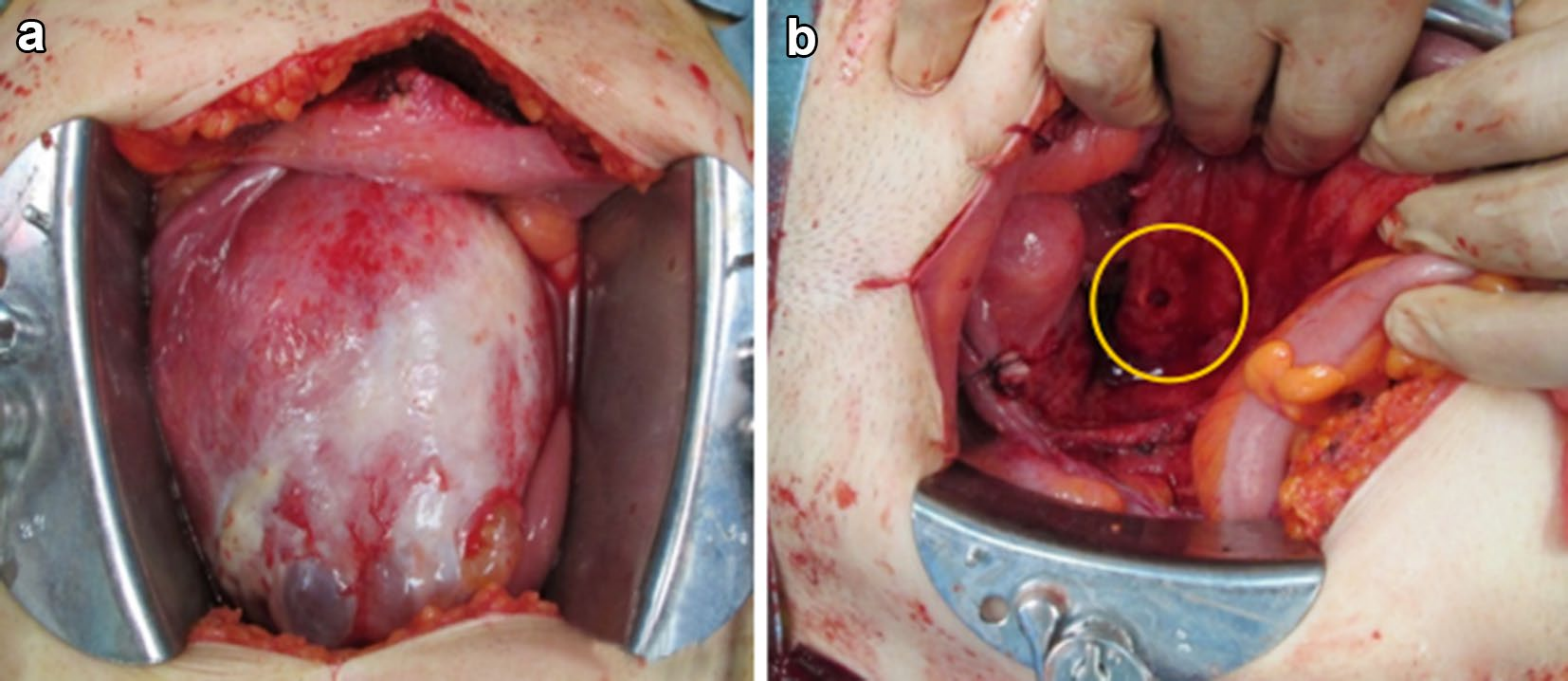
Surgical images. **a** An ovarian tumor with a smooth surface occupies the pelvic cavity. **b** After left salpingo-oophorectomy, a fistula of approximately 1 cm in diameter is seen on the surface of the rectum at the site of adhesion

**Fig. 5:**
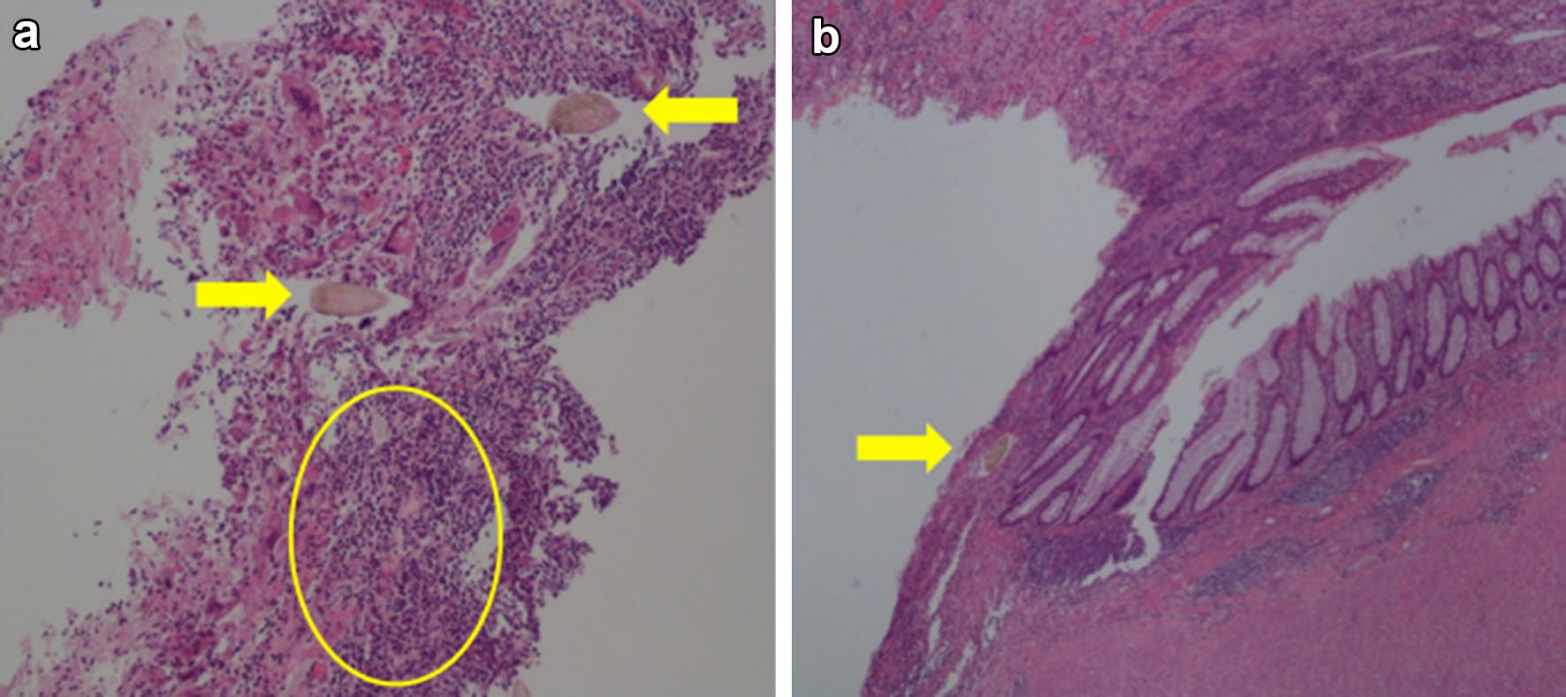
Histopathological images. **a** A histological section of the mature cystic teratoma reveals abundant inflammatory cell invasion (*circled section*) and hair-shaft tissue (*arrows*). **b** A section of the rectal-fistula site shows hair-shaft tissue in the serous membrane of the rectum (*arrow*)

Surgical findings were as follows. The left ovary was enlarged to approximately 10 cm in diameter and occupied most of the pelvic cavity. The ovarian tumor had a smooth surface, and part of the dorsal side was closely adhered to the rectum (Fig. 4a). The tumor was carefully separated from the rectum, and drainage of the tumor contents from the separation site revealed bright-green pus with a strong odor. After completion of the left salpingo-oophorectomy, the resected tissue was submitted for frozen-section pathological diagnosis during surgery. The resulting diagnosis was ovarian mature cystic teratoma accompanied by inflammatory granulation, with no malignant findings. In addition, a fistula of approximately 1 cm in diameter was observed in the anterior rectal wall at the site of separation from the tumor (Fig. 4b). Based on the result of frozen-section, it was possible to perform partial resection of rectal wall as minimal surgery, but necessity of histological examination of the tissue surrounding the rectal fistula was considered, and upper anterior resection of the rectum without temporary colostomy was performed at same time following the left salpingo-oophorectomy. Postoperative progress was satisfactory, and the patient was discharged on the 9th day after surgery. The final pathological diagnosis was identical to the pathological diagnosis during surgery, confirming a benign left-sided ovarian mature cystic teratoma. Findings of abundant inflammatory cell invasion in the cyst wall and hair-shaft tissue were consistent with a mature cystic teratoma (Fig. 5a). Further, although inflammatory cell invasion and hair-shaft tissue were observed in the rectal serosa, no structural disorders such as diverticulitis of the colon or signs of malignancy were observed (Fig. 5b).

## Discussion

Ovarian mature cystic teratomas are the most common ovarian tumor, comprising 10–25 % of all cases (Tandon et al. 2010). Multiple complications are known, of which the most common is torsion (16 % of tumors). Other reported complications include rupture (1–4 %), malignant transformation (1–2 %), and infection (1 %) (Park et al. 2008). Meanwhile, the formation of fistulas and invasion into the neighboring organs are extremely rare complications for this tumor, with frequency said to be less than 1 % (Tandon et al. 2010; von-Walter 2012).

The invasion of malignant cells into neighboring organs due to malignant transformation of the tumor is reported as the cause of fistula formation into the neighboring organs (Mitui et al. 1983; Okada et al. 2005; Song and Conner 2012; Gooneratne et al. 2015), but malignant transformation is not a necessary condition for the formation of fistulas (Kim et al. 1999).

In this instance, we performed surgery in consideration of the possible presence of a malignant tumor as the cause of fistula formation. Pathological diagnosis performed during surgery identified the tumor as a benign ovarian mature cystic teratoma. Histological examination of the area around the rectal fistula was deemed necessary to rule out the possibility of a malignant tumor, and an upper anterior resection of the rectum was performed. However, no signs of malignancy were found, and it was concluded that the fistula formation was due to inflammation. A retrospective review would suggest that the surgery conducted in this case (upper anterior resection of the rectum) was over-treatment, and trimming and restoration of the fistula site might have been sufficient. However, we believed that malignancy together with the invasion of malignant cells into neighboring organs was a necessary condition for the formation of fistulas.

Considering malignancy as a prerequisite for fistula formation into neighboring organs from ovarian mature cystic teratomas, we conducted a literature search and found 16 reported cases during the period from 1938 to 2015. A short review of these cases is shown in Table 1 together with the case we experienced. Of the 17 cases, fistula formation due to malignant transformation comprised only 4 cases (23.5 %), with inflammation as the actual cause in the majority of cases (13 cases, 76.5 %). However, one could still argue with a 23.5 % chance of malignancy that we performed an appropriate surgery. We may have been able to modify our policy once we received the pathological diagnosis during the surgery, but there is a risk of under-diagnosis.

**Table 1:** Reported cases of ovarian teratoma complicated with fistula formation

Author (date of publication)	Age	Symptoms	Involved organ	Surgical method	Cause of fistula formation
Robinson (1938)	64	Cystitis, dysuria	Bladder	USO, PC	Inflammation
Mitui et al. (1983)	72	Diarrhea containing hair	Sigmoid colon, small intestine	RS, colostomy, PRSI	Malignant transformation
Shiels et al. (1986)	21	Nausea, dyspareunia	Sigmoid colon	USO, fistulectomy, repair of the bowel defect	Inflammation
Ulstein et al. (1987)	30	Bladder stone	Bladder	USO, PC	Inflammation
Landmann et al. (1988)	22	Rectal bleeding	Rectum	USO, LAR	Inflammation
Suzuki et al. (1999)	64	Microscopic hematuria	Bladder, small intestine	TH, BSO, appendectomy, PRSI, PC	Inflammation
Tabata et al. (2004)	20	Pyuria	Bladder	USO, PC	Inflammation
Okada et al. (2005)	54	Abdominal pain, watery diarrhea	Small intestine	TH, BSO, PRSI, POM	Malignant transformation
Cebesoy et al. (2009)	30	Abdominal pain, purulent diarrhea	Rectum	USO, LAR	Inflammation
Tandon et al. (2010)	30	Pyuria, dysuria	Bladder	USO, PC	Inflammation
Rajaganeshan et al. (2011)	44	Weight loss, loose stools, rectal bleeding	Rectum	USO, repair of the bowel defect	Inflammation
Salame et al. (2011)	38	None	Sigmoid colon	RS	Inflammation
von-Walter et al. (2012)	25	Small bowel obstruction	Small intestine, transverse colon	USO, PRSI, repair of the bowel defect	Inflammation
Song et al. (2012)	73	Abdominal pain, constipation	Small intestine	USO, PRSI	Malignant transformation
Conway et al. (2012)	26	Abdominal pain, nausea	Transverse colon	Repair of the bowel defect	Inflammation
Gooneratne et al. (2015)	63	Abdominal distention, bloody diarrhea	Sigmoid colon	Exploratory laparotomy	Malignant transformation
Our case	43	Diarrhea	Rectum	USO, HAR	Inflammation

*USO* unilateral salpingo-oophorectomy, *BSO* bilateral salpingo-oophorectomy, *TH* total hysterectomy, *PC* partial cystectomy, *RS* recto-sigmoidectomy, *PRSI* partial resection of small intestine, *LAR* lower anterior resection, *HAR* high anterior resection, *POM* partial omentectomy

Shiels et al. (1986) consider the following steps to be involved in the pathogenesis of fistula formation: (1) rupture or perforation of the cyst, (2) small leakage from the cyst causing dense adhesion between the cyst and the surrounding organs, (3) circulatory disturbance of the walls of these structures due to the resultant necrosis and inflammation, and (4) formation of fistulas in the walls of these structures. In addition, they have mentioned that rupture or perforation of the cyst is likely caused by circulatory disorders or infection due to factors such as partial torsion, malignant transformation, and mechanical forces.

Fistula formation was observed in 20 sites in 17 patients, listed by organ as the urinary bladder (5 cases, 25.0 %), small intestine (5 cases, 25.0 %), sigmoid colon (4 cases, 20.0 %), rectum (4 cases, 20.0 %), and transverse colon (2 cases, 5.0 %). While Kim et al. (1999) have reported that fistulas are most likely to form in the urinary bladder, we consider their formation equally likely in any organ neighboring the tumor, or more likely to form in the lower gastrointestinal tract. Further, the symptoms presented will depend on the organ into which a fistula has formed.

Of the 13 cases in which inflammation was the cause, including the case we experienced, extended surgery appears to have been performed because of the possibility of malignancy in at least 5 cases (38.5 %). We too performed extended surgery in consideration of the possibility of a malignant tumor, but with the knowledge that inflammation is an extremely common cause of fistula formation from ovarian mature cystic teratomas, more limited surgery might have been possible, and we must reflect upon this point.

## Conclusions

Formation of fistulas into neighboring organs is an extremely rare complication of ovarian mature cystic teratomas. In the present case, we experienced the fistula formation into the rectum, and concerned about the possibility of malignancy, nevertheless no malignancy was observed. While opportunities to encounter such a case in clinical practice seldom arise, if a case is encountered or this complication is suspected, treating doctors must remember that the presence of malignant transformation is not a necessary condition for formation, and in fact inflammation is the cause in most cases. While surgical intervention is necessary when the formation of fistulas is observed, it should be performed subject to a thorough assessment of the possible causes and with due attention to the avoidance of over-treatment.
